# Combination of Lectures for Case-report Writing and Advances in Internet Technology May Be a Possible Solution for “Encouraging Students and Trainees to Write”

**DOI:** 10.31662/jmaj.2023-0045

**Published:** 2023-05-22

**Authors:** Yosuke Kakisaka, Chiharu Ota, Jun-Ichi Kameoka

**Affiliations:** 1 Department of Epileptology, Tohoku University Graduate School of Medicine, Sendai, Japan; 2 Department of Pediatrics, Tohoku University Hospital, Sendai, Japan; 3 Division of Hematology and Rheumatology, Tohoku Medical and Pharmaceutical University, Sendai, Japan

**Keywords:** Undergraduate, Lectures for case writing, Internet, You Tube, Google Docs

We read with interest the paper by Saeki emphasizing the importance of teaching scientific writing in medical education programs ^(1)^. He noticed that limited time is allocated to writing medical papers in Japanese medical schools although medical English writing is taught sufficiently.

He proposes incorporating the subject of “Medical Writing” into the medical school curriculum. Our work thus far has high affinity with his initiative. In 2013, we started a lecture titled “How to Write a Case Report” for 4^th^-year medical students of Tohoku University, the details of which have been described previously ^(2)^. This lecture was also started in Tohoku Medical and Pharmaceutical University in 2018. The case report was chosen as a format for teaching writing because for trainees and students, patients are the most familiar teachers, and case reports are easy to write because of their conciseness.

To motivate students and ensure comprehensibility of the lecture, various methods have been developed. For example, we introduce four writing steps that require clear objectives and specific actions. This “four-step division” is intended to reduce the writer’s workload by decreasing their tasks and clarifying what needs to be done at each stage in the process. These attempts proved to be successful as approximately 80% students reported that the lecture was useful and motivating ^(3)^. To make this effect more pervasive and sustainable, the lectures are archived and available for free on YouTube. We also share our ideas for improving academic writing education based on the challenges we faced during our education and a literature review of papers reporting delayed academic writing education in Japan ^(4)^. We also utilize Google Docs, which can enable real-time interactive writing and editing for students who are separated from instructors.

One of the best ways to motivate students to write is the publication of their first paper ^(5)^. Accomplished students can gain motivation for the next step of case-report writing, which is one of the most rewarding exercises ^(5)^. These students are taking the first step toward becoming independent researchers. With further case-report writing practice, students will be able to think and write independently about their clinical experiences.

Thus, we believe that the combination of organizing lectures for case-report writing and using advanced internet technologies will provide more opportunities for teaching and learning writing skills easily and efficiently.

## Article Information

### Conflicts of Interest

None

### Author Contributions

The authors are responsible for the conceptualization and the writing manuscript.

### Approval by Institutional Review Board (IRB)

Not applicable.
